# Applicability of in vivo staging of regional amyloid burden in a cognitively normal cohort with subjective memory complaints: the INSIGHT-preAD study

**DOI:** 10.1186/s13195-019-0466-3

**Published:** 2019-01-31

**Authors:** Fatemah A. Sakr, Michel J. Grothe, Enrica Cavedo, Irina Jelistratova, Marie-Odile Habert, Martin Dyrba, Gabriel Gonzalez-Escamilla, Hugo Bertin, Maxime Locatelli, Stephane Lehericy, Stefan Teipel, Bruno Dubois, Harald Hampel, for the INSIGHT-preAD study group, Hovagim Bakardjian, Habib Benali, Hugo Bertin, Joel Bonheur, Laurie Boukadida, Nadia Boukerrou, Enrica Cavedo, Patrizia Chiesa, Olivier Colliot, Bruno Dubois, Marion Dubois, Stéphane Epelbaum, Geoffroy Gagliardi, Remy Genthon, Marie-Odile Habert, Harald Hampel, Marion Houot, Aurélie Kas, Foudil Lamari, Marcel Levy, Simone Lista, Christiane Metzinger, Fanny Mochel, Francis Nyasse, Catherine Poisson, Marie-Claude Potier, Marie Revillon, Antonio Santos, Katia Santos Andrade, Marine Sole, Mohmed Surtee, Michel Thiebaud de Schotten, Andrea Vergallo, Nadjia Younsi, the Alzheimer Precision Medicine Initiative (APMI)

**Affiliations:** 1 Department of Psychosomatic Medicine, Clinical Dementia Research, Faculty of Medicine, Rostock University, Rostock, Germany; 2 German Center for Neurodegenerative Diseases (DZNE), Rostock, Germany; 3 AXA Research Fund and Sorbonne University Chair, Paris, France; 4 Sorbonne University, GRC n° 21, Alzheimer Precision Medicine (APM), AP-HP, Pitié-Salpêtrière Hospital, Boulevard de l’hôpital, F-75013 Paris, France; 5 Brain and Spine Institute (ICM), INSERM U 1127, CNRS UMR 7225, Boulevard de l’hôpital, F-75013 Paris, France; 6 Department of Neurology, Institute of Memory and Alzheimer’s Disease (IM2A), Pitié-Salpêtrière Hospital, AP-HP, Boulevard de l’hôpital, F-75013 Paris, France; 7 Qynapse, Paris, France; 8 Sorbonne University, UPMC University Paris 06, CNRS, INSERM, Laboratoire d’Imagerie Biomédicale, F-75013 Paris, France; 9 Multi-center Neuroimaging Platform, https://www.cati-neuroimaging.com; 10 Department of Nuclear Medicine, Pitié-Salpêtrière Hospital, AP-HP, F-75013 Paris, France; 11 Department of Neurology, University Medical Center of the Johannes-Gutenberg-University Mainz, Langenbeck str, 155131 Mainz, Germany; 12 Centre de NeuroImagerie de Recherche (CENIR), Institut du Cerveau et de la Moelle Epiniere (ICM), Paris, France; 13 Department of Neuroradiology, Salpêtriere Hospital, Paris, France

**Keywords:** Amyloid PET, In vivo staging, Subjective memory complaint

## Abstract

**Background:**

Current methods of amyloid PET interpretation based on the binary classification of global amyloid signal fail to identify early phases of amyloid deposition. A recent analysis of 18F-florbetapir PET data from the Alzheimer’s disease Neuroimaging Initiative cohort suggested a hierarchical four-stage model of regional amyloid deposition that resembles neuropathologic estimates and can be used to stage an individual’s amyloid burden in vivo. Here, we evaluated the validity of this in vivo amyloid staging model in an independent cohort of older people with subjective memory complaints (SMC). We further examined its potential association with subtle cognitive impairments in this population at elevated risk for Alzheimer’s disease (AD).

**Methods:**

The monocentric INSIGHT-preAD cohort includes 318 cognitively intact older individuals with SMC. All individuals underwent 18F-florbetapir PET scanning and extensive neuropsychological testing. We projected the regional amyloid uptake signal into the previously proposed hierarchical staging model of in vivo amyloid progression. We determined the adherence to this model across all cases and tested the association between increasing in vivo amyloid stage and cognitive performance using ANCOVA models.

**Results:**

In total, 156 participants (49%) showed evidence of regional amyloid deposition, and all but 2 of these (99%) adhered to the hierarchical regional pattern implied by the in vivo amyloid progression model. According to a conventional binary classification based on global signal (SUVR_Cereb_ = 1.10), individuals in stages III and IV were classified as amyloid-positive (except one in stage III), but 99% of individuals in stage I and even 28% of individuals in stage II were classified as amyloid-negative. Neither in vivo amyloid stage nor conventional binary amyloid status was significantly associated with cognitive performance in this preclinical cohort.

**Conclusions:**

The proposed hierarchical staging scheme of PET-evidenced amyloid deposition generalizes well to data from an independent cohort of older people at elevated risk for AD. Future studies will determine the prognostic value of the staging approach for predicting longitudinal cognitive decline in older individuals at increased risk for AD.

**Electronic supplementary material:**

The online version of this article (10.1186/s13195-019-0466-3) contains supplementary material, which is available to authorized users.

## Background

Amyloid PET imaging is considered a direct in vivo measure of cortical amyloid load with a high specificity and a relatively strong correlation between the in vivo amyloid signal in PET and the post-mortem quantification of neuritic amyloid plaque load [1–3]. Currently, the majority of studies use a binary classification of global amyloid signal into positive and negative categories. Several postmortem studies, however, suggest a relatively consistent pattern of sequential regional amyloid involvement, with initial amyloid accumulation in the associative neocortex, then spreading through the primary sensory-motor cortex and the medial temporal allocortex to subcortical regions (striatum, thalamus, and cholinergic basal forebrain) and finally to the brain stem and cerebellum [3–6]. Based on these findings, two recent studies explored the topographical pattern of amyloid spread in vivo using regional analysis of amyloid PET signal in cognitively normal (CN), mild cognitive impairment (MCI), and Alzheimer’s disease (AD) individuals, which revealed highly consistent results with the findings described by post-mortem studies [6, 7].

Grothe et al. further tested the utility of this progression model for staging of individual deposition patterns [7]. They found that the individual deposition patterns closely adhered to the regional hierarchy implied by the progression model, allowing them to classify over 95% of participants with detectable regional amyloid deposition into one of four successive amyloid stages. Although the earliest in vivo amyloid stages were mostly missed by conventional binary classification approaches based on global amyloid signal, they were associated with significantly reduced cerebrospinal fluid (CSF) Aβ42 levels, corroborating the pathologic origin of these PET signal elevations [7]. Moreover, advanced in vivo amyloid stages were most frequently observed in cognitively impaired patients (MCI or AD dementia) and correlated with cognitive deficits in healthy elderly individuals [7].

The primary goal of the current study was to explore the validity of this recently proposed in vivo staging scheme [7] and its association with cognitive function in independent data from a large cohort of cognitively intact older individuals with subjective memory complaints, the “INSIGHT-preAD” cohort [8–10].

## Methods

### Participants

All the data for this project were collected for the INSIGHT-preAD study which is a mono-centric academic university-based cohort derived from the Institute for Memory and Alzheimer’s Disease (IM2A) at the Pitié-Salpêtrière University Hospital in Paris, France, with the objective of investigating the earliest preclinical stages of AD and its development including influencing factors and markers of progression [11].

The INSIGHT-preAD study includes 318 cognitively normal Caucasian individuals from the Paris area, between 70 and 85 years old, with subjective memory complaints and with defined brain amyloid status. The study aims at 7 years of follow-up, with the 2-year follow-up being completed in 2017. Demographic, cognitive, functional, nutritional, biological, genetic, genomic, imaging, electrophysiological, and other assessments were performed at baseline. Subjective memory complaints were confirmed by an affirmative answer to both of the following questions: (i) “Are you complaining about your memory?”, and (ii) “Is it a regular complaint which lasts more than 6 months?”

Demographic characteristics, including cognitive performance and ApoE genotype, are shown in Table 1. Each participant had a total recall at the Free and Cued Selective Reminding Test in the normal range (mean 46.1 ± 2.0). Written informed consent was provided by all participants. The study was approved by the local Institutional Review Board and has been conducted in accord with the Helsinki Declaration of 1975.

**Table 1:** Summary of participants’ demographics

	*N*	Age (years)	Gender (F/M)	ApoE-ε4	MMSE score (0–30)	Education (0–8)
Amyloid +ve	68	76.6 ± 3.6	44/24	38.2%	28.5 ± 0.91	6.0 ± 2.1
Amyloid −ve	250	75.9 ± 3.5	157/93	12.8%	28.7 ± 0.96	6.2 ± 2.0
All subjects	318	76.1 ± 3.5	201/117	18.2%	28.67 ± 0.95	6.2 ± 2.0

The table presents the demographic features among the whole INSIGHT-preAD cohort as well as the distribution within two main categories, amyloid-positive and amyloid-negative as classified using the conventional threshold of SUVR = 1.10 applied to global 18F-florbetapir PET signal intensity normalized to the average signal in the whole cerebellum [12, 13]. Data are mean values ± standard deviation

*N* number of participants in each category, *ApoE-ε4* percent of participants positive for ε4 allele, *MMSE* Mini Mental State Examination

### Cognitive tests

A comprehensive neuropsychological battery was administered to all participants of the INSIGHT-preAD cohort including the Mini-Mental State Examination (MMSE) [14] to asses global cognition, the Free and Cued Selective Reminding Test (FCSRT) and Memory Binding Test (MBT) [15–17] for episodic memory, Letter and Category Verbal Fluency test [18–20] for instrumental and executive functions, the Rey-Osterrieth Complex Figure Copy [21] for visuo-spatial abilities; Digit span (forward and backward) [22, 23], the Trail Making Test (TMT) [24], and the Frontal Assessment Battery [25] for the assessment of working memory and executive function. In order to reduce the high dimensionality of the detailed cognitive test data, we ran a principal component analysis (PCA) on standardized *z*-scores of the 15 available cognitive test scores to sum up the covariates into representative eigenvectors. However, we excluded from the PCA analysis the scores of MMSE, Frontal Assessment Battery, and total (free and cued) delayed recall of FCSRT (index test for study inclusion) due to lack of variance in their scores among participants.

### ApoE genotype

DNA was prepared from frozen blood samples using the 5Prime Archive Pure DNA purification system according to the manufacturer’s instructions. ApoE genotypes were determined for each individual using PCR-based Sanger sequencing. Exon 4 from ApoE gene containing the SNP corresponding to the ε3/ε4 alleles was amplified using PCR with the following primers: ApoE sense, 5′-TAAGCTTGGCACGGCTGTCCAAGGA-3′; ApoE antisense, 5′-ACAGAATTCGCCCCGGCCTGGTACAC-3′. For each sample, the reaction mixture (50 μl) contained 200 ng of genomic DNA, 10 μl PCR Flexi buffer (5×), 3 μl MgCl2 (25 mM), 1 μl dNTPs (10 mM), 1 μl of each forward and reverse primers (10 μM), and 0.25 μl GO Taq DNA polymerase (Promega). The cycling program was carried out after a preheating step at 95 °C for 2 min and 35 cycles of denaturation at 95 °C for 1 min, annealing at 68 °C for 1 min and extension at 72 °C for 1 min. The amplified fragments were then purified and sequenced using the same primers [11].

### Imaging data acquisition

In the Pitié-Salpêtrière University Hospital in Paris, all the amyloid PET scans were acquired in a single session on a CT-PET scanner (Gemini GXL, Philips, Cleveland, USA) 50 ± 5 min after the injection of approximately 370 MBq (333–407 MBq) of 18F-florbetapir (AVID radiopharmaceuticals). PET acquisition consisted of 3 × 5-min frames, in a 128 × 128 acquisition matrix, with a voxel size of 2 × 2 × 2 mm^3^.

Images were then reconstructed using the iterative LOR-RAMLA algorithm (10 iterations). Reduction of noise was modulated by the relaxation parameter lambda, which was set to 0.7. All corrections (attenuation, scatter, and random coincidence) were integrated in the reconstruction [26]. The reconstructed PET image resolution was 7.5 mm FWHM.

MRI scans were acquired on a Siemens Verio 3 T scanner at the CENIR in the Brain and Spine Institute, Paris, France. A T1-weighted image was acquired using a fast three-dimensional gradient echo pulse sequence using a magnetization preparation pulse (Turbo FLASH) and with the parameters of TR = 2300 ms; TE = 2.98 ms; IT = 900 ms; flip angle = 9°; 1-mm isotropic voxel size; matrix 256 × 240; bandwidth 240 Hz/Px [26].

### Imaging data pre-processing

Images were preprocessed using Statistical Parametric Mapping software version 12 (SPM12) (The Wellcome Trust Centre for Neuroimaging, Institute of Neurology, University College London) implemented in Matlab 2013. The pre-processing pipeline followed the routine previously described in Grothe et al. [7]. First, each subject’s averaged PET frames were co-registered to their corresponding T1-weighted MRI scan. Then, partial volume effects (PVE) were corrected in native space using the three-compartmental voxel-based post-reconstruction method as described by Müller-Gӓrtner and colleagues (MG method) [27]. The corrected PET images were spatially normalized to an aging/AD-specific reference template using the deformation parameters derived from the normalization of their corresponding MRI. The pre-processing pipeline is summarized in the schematic diagram provided in Additional file 1: Figure S1.

The regional 18F-florbetapir PET mean uptake values were estimated for 52 brain regions defined by the Harvard–Oxford structural atlas [28], including both cortical and subcortical regions (https://fsl.fmrib.ox.ac.uk/fsl/fslwiki/Atlases). Standard uptake value ratios (SUVRcereb) were computed for the 52 brain regions by dividing the mean uptake values by the mean uptake value of the whole cerebellum as estimated in non-PVE-corrected PET data [7, 29–31].

In accordance with the methods used for the published PET-based amyloid staging approach, we based the cutoff used for determining regional amyloid positivity on a cutoff value of SUVRcereb = 1.135 [7], which lies in between the two most widely used global signal cutoffs for non-PVE-corrected 18F-florbetapir PET SUVRs, i.e., SUVRcereb = 1.10 [12, 13, 32], which represents the upper limit of observed signal in a group of healthy controls, and SUVRcereb = 1.17, which corresponds to the lowest signal observed in a group of AD dementia patients [33, 34]. This threshold was converted to PVE-corrected data employed in the regional staging approach using linear regression. Thus, global 18F-florbetapir PET uptake mean values within a cortical composite mask were calculated on PET data both corrected and non-corrected for PVE and these were scaled to the mean signal of the whole cerebellum (extracted from non-PVE-corrected data). Global 18F-Florbetapir PET SUVRcereb of both non-corrected (*X*-axis) and PVE-corrected PET (*Y*-axis) data were plotted, and linear regression analysis indicated a very strong correlation between the two values (*R* = 0.94). The linear regression equation was used to transform the mean cutoff value of SUVRcereb = 1.135 to a value of SUVRcereb = 0.98 in the PVE-corrected PET data used in our present study [7] (Additional file 2: Figure S2).

### Data analysis

#### Individual staging of amyloid deposition according to previously reported four-stage model

We projected our regional SUVRcereb values on the previously published four-stage model of amyloid pathology progression derived from 18F-Florbetapir PET data of cognitively normal older individuals enrolled in the Alzheimer’s disease Neuroimaging Initiative (ADNI) study [7]. This four-stage model was estimated by counting the frequency of amyloid positivity across the 52 brain regions defined in the Harvard–Oxford structural atlas and then merging the regions into four broader anatomical divisions based on equal proportions of the observed range of involvement frequencies. The four anatomical divisions defining the staging scheme are illustrated in Additional file 3: Figure S3, and full details on the development of this staging approach are provided in the original publication [7].

Following the approach described in [7], an anatomical division was considered positive for amyloid pathology if at least 50% of the regions included in this division exceeded the cutoff value (SUVRcereb = 0.98) in the respective participant. Subsequently, participants were classified as stage I if only the first division was considered positive. Then, the successive stages II–IV were defined by the additional involvement of their corresponding divisions 2, 3, and 4, respectively. Participants who exhibited amyloid positivity in any division without concurrent amyloid positivity in the preceding divisions were classified as non-stageable (mismatch)*.*

For comparison, 18F-Florbetapir PET scans were also conventionally classified into global amyloid-positive or amyloid-negative categories based on a commonly used cutoff of SUVRcereb > 1.10, applied to the global composite SUVRCereb values (non-PVE-corrected).

#### Reproducibility of the amyloid progression model

In order to assess the reproducibility of the regional progression model underlying the hierarchical staging scheme, we re-estimated the model by calculating the regional frequency of amyloid positivity across the 18F-Florbetapir PET scans of the INSIGHT-preAD cohort. Correspondence between the model derived from the INSIGHT-preAD cohort and the original model derived from the ADNI cohort was assessed quantitatively using the Spearman correlation between the respective ranks of the 52 studied brain regions.

### Statistical analysis

All the data were statistically analyzed using the SPSS Statistics software package, version 23.0, developed by IBM. An association between in vivo amyloid stage and ApoE-ε4 allele frequency was assessed using chi-squared (χ^2^) test. Analysis of covariance (ANCOVA) was used to examine the covariation between amyloid stage and scores of the cognitive principal components, as well as the cognitive tests scores being most representatives for each of these components, while adjusting for the covariates age and gender. For comparison, we also applied the ANCOVA analysis to the conventional binary amyloid status. *P* values were corrected for multiple comparisons using the Bonferroni correction.

## Results

### Individual staging based on hierarchical four-stage model of regional amyloid deposition

**Fig. 1:**
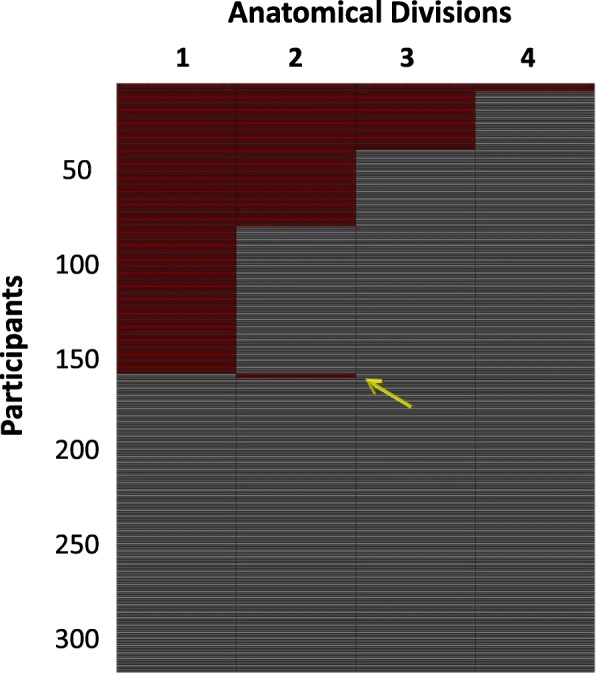
Individual staging of INSIGHT-preAD cohort participants based on regional amyloid burden. This figure shows the individual staging of the INSIGHT-preAD cohort, where each row represents a participant in the study while the columns represent the 4 anatomical divisions. The red and the gray colors denote presence and absence of amyloid, respectively, in each anatomical division. The yellow arrows point to mismatching individuals

The individual staging of INSIGHT-preAD participants based on regional amyloid burden is displayed in Fig. 1. One hundred fifty-six participants (49%) showed evidence of regional amyloid deposition, and only two of these (1.3%) were found to violate the proposed regional hierarchy implied by the four-stage model, providing evidence for the consistency of this stage model across different cohorts. Both mismatching individuals were found to be positive for the second anatomical division while lacking amyloid positivity for the regions of the first anatomical division. Among the five regions comprising the first anatomical division, the mismatching individuals exhibited positivity for the inferior temporal gyrus (both anterior and posterior divisions) while lacking amyloid deposition in the remaining regions, namely anterior cingulate gyrus, temporal fusiform cortex, and parietal operculum.

Exploring the distribution of the different stages of the model against the conventional binary classification model (based on a global signal threshold of SUVRcereb > 1.10), we observed that almost all the subjects in stage III or IV (96.8% and 100%, respectively) were classified as amyloid-positive. By contrast, almost all the subjects who belonged to stages 0 and I were classified as amyloid-negative (98.1% and 98.7%, respectively). Moreover, about 30% of individuals in stage II were classified as amyloid-negative (Table 2). When matching the global signal cutoff to the cutoff used for determining regional positivity (SUVRcereb = 1.135), the percentage of negatively classified individuals in stage II rose to 40%.

**Table 2:** Comparing individual amyloid stages to conventional binary amyloid status

	Stage 0	Stage 1	Stage 2	Stage 3	Stage 4
SUVR > 1.1	3 (1.9%)	1 (1.3%)	29 (72.5%)	31 (96.9%)	4 (100%)
SUVR > 1.135	0 (0%)	0 (0%)	24 (60%)	31 (96.9%)	4 (100%)
Number of subjects	162	78	40	32	4

The table presents the distribution of the stages of the in vivo amyloid staging model among the INSIGHT-preAD participants compared to conventional binary amyloid status. Data represents the number of participants with global cortical measure exceeding the cutoff of (SUVR = 1.1) and cutoff of (SUVR = 1.135), respectively, and their percentage among the total participants comprising the respective stage

### Association of amyloid stage with APOE genotype and cognitive performance

In vivo amyloid stage was significantly associated with ApoE-ε4 status, such that the percentage of ApoE-ε4 carriers increased with increasing amyloid stage (chi-squared (χ^2^) test, *p* = 0.001) (Table 3).

**Table 3:** Amyloid progression model stages and ApoE-ε4 status

	N	Stage 0	Stage 1	Stage 2	Stage 3	Stage 4
ApoE-ε4 (+ve)	58	17 (10.5%)	12 (15.4%)	15 (37.5%)	13 (40.6%)	1 (25.0%)
ApoE-ε4 (−ve)	258	145 (89.9%)	66 (84.6%)	25 (62.5%)	19 (59.4%)	3 (75.0%)
All subjects	316	162	78	40	32	4

The table presents the distribution of stageable participants (316 out of 318) in the INSIGHT-preAD cohort among the in vivo amyloid stages and their corresponding ApoE-ε4 status

*N* number of participants in each category, *ApoE-ε4* apolipoprotein E (ε4 allele)

The principal component analysis of the cognitive tests, including memory, executive, and attention functions, identified three main components that accounted for 45.5% of the variance in the data (Additional file 4: Table S1). The highest loading in the first component was for FCSRT “Total free recall scores” and in the second component for the MCT “Immediate Total Free Recall List 1 and 2.” The third component mainly represented tests of executive and attention functions and showed highest loadings on “TMT-B scores.”

In vivo amyloid stage was not significantly associated with any of the principal component scores, but showed relatively weak effects on FCSRT total recall scores (*p* = 0.022, partial *η*^2^ = 0.063) and TMT-B scores (*p* = 0.036, partial *η*^2^ = 0.056), which did not survive correction for multiple comparisons. Moreover, the effects appeared to be primarily driven by low cognitive scores of the few participants in amyloid stage IV (*N* = 4) and did not remain (nominally) significant when these participants were removed. The binary conventional approach had no significant effect on any of the three principal components or their most representative individual tests scores (full statistics for all tests and plots of the data are reported in Additional file 5: Table S2).

### In vivo amyloid progression model based on frequency of regional involvement

In the INSIGHT-preAD cohort, the inferior temporal gyri showed the highest frequency of involvement (about ~ 90%) followed by the lateral occipital cortices and middle temporal gyri (~ 70% and ~ 60% respectively) and the other associative cortex regions. The precuneus cortex and the cingulate gyri surprisingly showed an intermediate frequency of involvement (~ 20%) which was rather close to the primary sensory-motor regions (~ 15–20%). The brain areas less involved were the striatum and the parahippocampal regions (~ 2–5%), while no regional amyloid pathology was detected in the thalamus and hippocampus (Additional file 6: Figure S4).

**Fig. 2:**
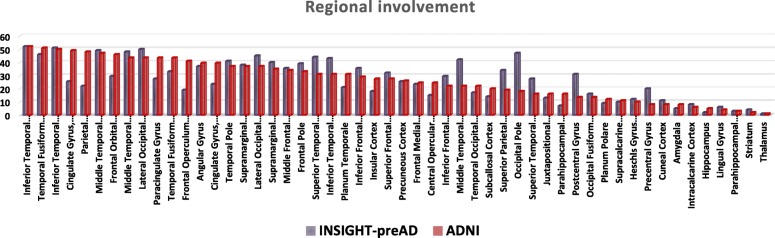
Comparison of the in vivo amyloid progression model derived from the INSIGHT-preAD cohort versus the original model. This figure displays the ranks of the 52 studied brain regions according to the re-estimated amyloid progression model in the INSIGHT-preAD cohort (violet bars) and directly compares them to the ranks in the originally estimated model in the ADNI data (red bars)

Figure 2 compares the ranks of the 52 studied brain regions between the estimated progression model in the INSIGHT-preAD cohort and the originally estimated model in the ADNI data [7]. Overall, the two models showed a relatively good correspondence with a Spearman correlation between the respective ranks of *R* = 0.75 (*p* < 0.001). Both models generally agreed on a pattern of involvement frequencies that are highest in the inferior temporal lobe and other heteromodal association areas, intermediate in several primary sensory-motor regions (e.g., precentral gyrus and cuneal cortex), and lowest in the medial temporal lobe and subcortical areas. However, some discrepancies between both models were also notable. For example, the INSIGHT-preAD cohort showed a relatively higher frequency of involvement in posterior (occipital and parietal) brain regions and the lateral temporal lobe (middle and superior temporal gyri), whereas the anterior and posterior cingulate gyri, fronto-orbital and opercular regions were relatively less frequently involved.

## Discussion

Here, we adopted a hierarchical region based in vivo amyloid staging model proposed recently [7] and applied it on a large independent cohort of older individuals at elevated risk for AD [8, 10]. Our findings provide evidence for the applicability of the in vivo amyloid staging model proposed by Grothe and colleagues to different cohorts. Indeed, 99% of the 156 individuals with detectable amyloid load in the INSIGHT-preAD cohort adhered to the sequential regional pattern of the model. Moreover, it allowed for identifying 49% of clinically normal older individuals as having evidence of regional amyloid deposition in this preclinical cohort opposed to the 21.5% identified as being amyloid-positive using the conventional global measure. Almost all individuals classified as stage I and about 30% of individuals classified as stage II according to the regional staging approach were considered amyloid-negative according to the conventional global measure. Both stages correspond to Thal amyloid phase 1 denoting amyloid deposits confined to the associative neocortex [5]. Thus, in vivo amyloid staging could have crucial implications for clinical trials, as it may allow the identification of individuals at the very early stages of disease pathogenesis.

These findings are consistent with the results of previous studies exploring the correspondence between Thal amyloid staging at autopsy and the ante-mortem depicted PET signal using different radioisotope ligands, namely C^11^ PiB and F^18^ radiolabeled ligands [2, 3, 6]. Some studies concluded that amyloid PET scans are particularly effective in detecting advanced Thal phases and that the early Thal phases (0–2) with amyloid deposition confined to the neocortex were always associated with a negative scan [3, 35, 36]. Analogously, Murray et al. suggested that PET positivity assigned based on global PET signal and the currently used thresholds fail to recognize initial amyloid phases [2]. They showed that a cutoff point of 1.4 SUVR on ante-mortem PiB PET normalized to whole cerebellar signal corresponded to Thal amyloid phase 2 at autopsy [2, 36]. However, Ikonomovic et al. compared the regional CERAD neuritic plaque score at autopsy with ante-mortem PET scans and suggested that 18F-flutemetamol PET may detect amyloid-β plaques early in neocortex even below the level usually associated with clinically significant (moderate) burden [3]. Subsequently, they recommended modifying the amyloid PET scans interpretation method to fit the targeted clinical setting, using a more sensitive method for identifying at risk subjects with still preserved cognitive functions [3]. Consequently, many studies recommended using a lower cutoff than the currently used to assign amyloid positivity based on global PET signal [2, 37]. This approach, however, increases the risk of false positive findings. The solution proposed by the regional staging approach is to consider detailed topographical differences between individuals to identify the early amyloid accumulators who are usually misclassified as negative for amyloid [2], while minimizing misclassification of low unspecific binding.

The second goal of our study was to explore the association between amyloid stage and cognitive performance in this cohort of cognitively intact individuals with subjective memory complaints. We included in our analysis scores of neuropsychological tests that assess the various disease-specific cognitive domains, such as episodic memory and memory binding, executive functions, processing speed and attention [38–42]. Overall, associations between cognitive performance and amyloid load showed only small and non-significant effects, regardless of whether amyloid load was assessed using in vivo amyloid stage or conventional binary amyloid status. This could be attributed to the limited variability of the cognitive test scores in a cognitively normal performing cohort. In subsequent follow-up data from the INSIGHT-preAD cohort, we will be able to determine whether regional amyloid stages associate with a stage-proportional risk for longitudinal cognitive decline, thus potentially providing more fine grained risk stratification compared with the binary classification of global amyloid load [43]. The lack of associations between episodic memory and amyloid load in our cohort contradicts previous studies that found that subtle episodic memory changes occur in early, preclinical stages of the disease [39, 44–48]. This contradiction may be attributable to the inclusion criteria of the INSIGHT-preAD cohort requiring normal performance in the FCSRT total recall scores, thus restricting the variability in episodic memory performance in this cohort and possibly masking its cross-sectional association with amyloid burden.

In a secondary analysis, we also assessed the reproducibility of the regional amyloid progression model underlying the hierarchical staging scheme by calculating the regional frequency of amyloid positivity across the 18F-florbetapir PET scans of the INSIGHT-preAD cohort. Overall, the re-estimated model in the INSIGHT-preAD cohort showed a relatively good correspondence with the originally estimated model in the ADNI data. Both models agreed on a general pattern of involvement frequencies that are highest in the inferior temporal lobe and other heteromodal association areas, intermediate in several primary sensory-motor regions and lowest in the medial temporal lobe and subcortical areas. This pattern also largely agrees with regional involvement frequencies observed in a previous study using 18F-florbetaben PET data [6] and is consistent with long-standing neuropathologic estimates of regionally progressing amyloid pathology [5, 49]. However, besides the relatively good overall correspondence, on a regionally more detailed level, some notable discrepancies were also evident between the models derived from the INSIGHT-preAD and ADNI cohorts (Fig. 2), which may relate to differences in the specific characteristics of both cohorts. Thus, the INSIGHT-preAD cohort is a highly selected mono-centric cohort of very old seniors who lack any objective cognitive decline despite presenting with subjective memory complaints. Due to the advanced age, a high prevalence of co-morbid pathologies, particularly cerebrovascular disease and cerebral amyloid angiopathy (CAA), can be expected and these may interact with the regional patterns of amyloid deposition. For example, CAA has been reported in up to 57% of individuals over 70 years and affects primarily the occipital and parietal lobes [50, 51]. Hence, the relatively higher involvement of posterior (occipital and parietal) over frontal brain regions in the INSIGHT-preAD data may potentially be explained by a higher prevalence of CAA in this relatively old cohort. On the other hand, the preserved cognitive performance of these individuals points to a higher brain resilience that may provide a relative resistance to regional pathology progression [52, 53]. Due to these specific cohort characteristics, a regional progression model derived from the INSIGHT-preAD cohort may not generalize well to the broader population of older people. However, it is notable that the regional differences in amyloid distribution in the INSIGHT-preAD data did not translate into an increased number of mismatching individuals in this cohort. Thus, overall amyloid deposition patterns across the four larger anatomical divisions considered in the original staging scheme based on ADNI data also showed a very consistent regional hierarchy across INSIGHT-preAD individuals. This highlights the potential of the proposed staging approach to provide a consistent staging of an overall amyloid progression pattern across four larger anatomical systems, while accounting for inter-individual differences in amyloid deposition at a higher regional resolution.

It is important to note that the proposed in vivo amyloid staging approach relies on a range of methodologic conditions that may affect the final staging outcomes, including for example the employed radiotracer (18F-Florbetapir), the methods used for signal quantification (PVE-corrected SUVR values) and for defining brain regions (Harvard-Oxford atlas), as well as the methods and thresholds used to define amyloid positivity for a region and for an anatomical division. While outside the scope of the present study, it would be important in future methodological studies to analyze in more detail the differential effects that these methodical choices may have on the staging outcomes and which methods may be the most accurate when compared to neuropathologic data as the gold standard.

For example, the described in vivo amyloid staging approach relies on PVE-corrected SUVR values for regional PET signal quantification [7]. While SUVR values are by far the most widely used metric for 18F-florbetapir PET scans [33] and for amyloid PET imaging in large-scale cohort studies with high-throughput PET scanning in general [26, 54–57], they are also known to lead to biased estimates when compared to the gold standard estimates derived from tracer kinetic modeling using dynamic PET acquisitions (i.e. BP_ND_ or DVR values) [58–60]. Thus, using DVR values, particularly arterial input-based values, could be an interesting option to further refine the in vivo amyloid staging model in future research.

Partial volume effect correction has been shown to enhance the accuracy of regional amyloid PET signal quantification and allows for inter-regional quantitative comparison as it alleviates the differential impact of partial volume effects on different brain regions [61, 62]. In healthy populations, it was described a decline in the global SUVR values following partial volume effect correction due to the predominant spill-in effects from the WM to the GM particularly in case of low brain atrophy and when using 18F-labeled amyloid tracers characterized by high non-specific binding to WM [63–65]. Another advantage of applying partial volume correction on PET data is that it enhances the sensitivity of detecting small changes in follow-up studies as it attenuates the bias induced by the concomitantly progressing cortical atrophy leading to underestimation of the SUVR in non-corrected PET data [29, 62, 63, 66]. This will enable us to further study the amyloid deposition model longitudinally, explore the transition rates between the identified stages of amyloid deposition, and study the correlation between the rates of transition and the rates of cognitive decline in the upcoming follow up data of the INSIGHT-preAD cohort.

For detection of regional amyloid-positivity the in vivo staging approach applies a constant threshold to all brain regions. This threshold is based on the mean value of the two most widely used cutoffs for defining amyloid-positivity based on global 18F-florbetapir PET SUVRs (SUVRcereb = 1.10 [12, 13, 32] and 1.17 [33, 34], respectively), which is further extrapolated to the PVE-corrected PET data used in the staging approach [7]. Grothe et al. used this relatively high and more conservative threshold owing to a potentially higher signal to noise ratio depicted when exploring the PET signal on a detailed regional level compared to the global PET measure. When estimating the global PET signal, the signal is averaged across all the regions comprising the global mask. Thus, in early amyloid deposition phases, when not all the included regions have already accumulated amyloid, the overall global signal may lie below the conventionally used thresholds, resulting in an amyloid-negative classification although considerable amyloid load may have already deposited in specific regions of the neocortex. However, using a fixed regional threshold for assigning regional amyloid-positivity regardless of differences in gray matter density and surface area between the different brain regions is a potential limitation of the described in vivo staging approach and an important methodological aspect to be further investigated. However, Grothe et al. conducted sensitivity analyses across the range of values between the two widely used cutoffs to confirm reproducibility of the amyloid deposition model in normal healthy individuals of the ADNI cohort across the entire range of amyloid cutoffs, suggesting relatively little inter-regional variability in noise levels in the PVE-corrected PET signal [7]. In contrast to the approach used by Grothe et al. [7], Cho et al. in their study determined regional amyloid-positivity using *Z* scores based on an older, globally negative, control population and a cutoff of *Z* score > 2.5 [6]. While the use of such region-specific thresholds may potentially account better for regionally differing noise levels, the definition of these thresholds will depend on the specific control cohort used, which limits the transferability of this approach and the generalization of the study results to other cohorts.

## Conclusions

In conclusion, our results support the validity and reproducibility of the in vivo staging model of regionally progressing amyloidosis in an independent preclinical cohort at elevated risk for AD. Further evaluation of the staging approach in parallel with longitudinal multi-domain cognitive performance will be crucial for assessing its prognostic value for predicting cognitive decline along the course of the disease.

## Additional files

Additional file 1: **Figure S1.** Schematic diagram summarizing the pre-processing pipeline. (PDF 276 kb)

Additional file 2: **Figure S2.** Regional amyloid positivity cutoff value estimation. The figure shows the linear regression plot of the Global 18F-florbetapir PET SUVRcereb of both non-corrected (X-axis) and PVE-corrected PET (Y-axis) along with the generated equation that was used to transform the regional cutoff value of SUVRcereb = 1.135 to a value of SUVRcereb = 0.98 in the PVE-corrected PET data. (PDF 190 kb)

Additional file 3: **Figure S3.** Model of the hierarchical in vivo amyloid staging scheme. This figure shows the 52 brain regions merged into four larger anatomical divisions based on equal partitions of frequency range as initially defined in the original model. Then the resulting amyloid progression stage (I-IV) is defined by the involvement of the corresponding anatomical division displayed in red in addition to the affected areas of the previous stage (displayed in blue). The amyloid progression stages are displayed on left, midline sagittal and basal brain views. (PDF 312 kb)

Additional file 4: **Table S1.** Principal component analysis applied on the neurocognitive test scores. The table shows the three main components that could be identified based on the principal component analysis and subsequently the contributing tests in each component. (PDF 97 kb)

Additional file 5: **Table S2.** Associations between in vivo amyloid stage and cognitive performance. Analysis of Covariance (ANCOVA) assessing the effect of amyloid stage and conventional binary amyloid status on scores of the main principal components as well as the most representative tests for each of these components. (PDF 240 kb)

Additional file 6: **Figure S4.** Amyloid progression model in the INSIGHT-preAD data. This figure shows the amyloid progression model in the INSIGHT-preAD data as implied by the frequency of involvement of the 52 studied brain regions. The frequencies were displayed on left, midline sagittal and basal brain views. (PDF 252 kb)

## Abbreviations

AD: Alzheimer’s disease
ADNI: Alzheimer’s Disease Neuroimaging Initiative
ANCOVA: Analysis of covariance
ANOVA: Analysis of variance
ApoE, ApoE-ε4: Apolipoprotein E, ε4 allele
Aβ, Aβ42: β-Amyloid, β amyloid peptide 1–42
C^11^ PiB: [^11^C] Pittsburgh Compound B
CAA: Cerebral amyloid angiopathy
CERAD: Consortium to Establish a Registry for Alzheimer’s disease
CN: Cognitively normal individuals
CSF: Cerebrospinal fluid
DVR: Distribution volume ratios
FCSRT: Free and Cued Selective Reminding Test
GM: Gray matter
MBT: Memory Binding Test
MCI: Individuals with mild cognitive impairment
MMSE: Mini-mental State Examination
MNI: Montreal Neurological Institute
PCA: Principal component analysis
PET: Positron emission tomography
PVE: Partial volume effects
SMC: Subjective memory complaints
SUVR: Standardized uptake value ratio
SUVRcereb: Standard uptake value ratios scaled by mean uptake value of the whole cerebellum
TMT: Trail Making Test
WM: White matter
