# Correction: Applicability of in vivo staging of regional amyloid burden in a cognitively normal cohort with subjective memory complaints: the INSIGHT-preAD study

**DOI:** 10.1186/s13195-022-01025-4

**Published:** 2022-09-14

**Authors:** Fatemah A. Sakr, Michel J. Grothe, Enrica Cavedo, Irina Jelistratova, Marie-Odile Habert, Martin Dyrba, Gabriel Gonzalez-Escamilla, Hugo Bertin, Maxime Locatelli, Stephane Lehericy, Stefan Teipel, Bruno Dubois, Harald Hampel, for the INSIGHT-preAD study group, Hovagim Bakardjian, Habib Benali, Hugo Bertin, Joel Bonheur, Laurie Boukadida, Nadia Boukerrou, Enrica Cavedo, Patrizia Chiesa, Olivier Colliot, Bruno Dubois, Marion Dubois, Stéphane Epelbaum, Geoffroy Gagliardi, Remy Genthon, Marie-Odile Habert, Harald Hampel, Marion Houot, Aurélie Kas, Foudil Lamari, Marcel Levy, Simone Lista, Christiane Metzinger, Fanny Mochel, Francis Nyasse, Catherine Poisson, Marie-Claude Potier, Marie Revillon, Antonio Santos, Katia Santos Andrade, Marine Sole, Mohmed Surtee, Michel Thiebaud de Schotten, Andrea Vergallo, Nadjia Younsi, the Alzheimer Precision Medicine Initiative (APMI), Lisi Flores Aguilar, Claudio Babiloni, Filippo Baldacci, Norbert Benda, Keith L. Black, Arun L. W. Bokde, Ubaldo Bonuccelli, Karl Broich, René S. Bun, Francesco Cacciola, Juan Castrillo, Enrica Cavedo, Roberto Ceravolo, Patrizia A. Chiesa, Olivier Colliot, Cristina-Maria Coman, Jean-Christophe Corvol, Augusto Claudio Cuello, Jeffrey L. Cummings, Herman Depypere, Bruno Dubois, Andrea Duggento, Stanley Durrleman, Valentina Escott-Price, Howard Federoff, Maria Teresa Ferretti, Massimo Fiandaca, Richard A. Frank, Francesco Garaci, Remy Genthon, Nathalie George, Filippo S. Giorgi, Manuela Graziani, Marion Haberkamp, Marie-Odile Habert, Harald Hampel, Karl Herholz, Eric Karran, Seung H. Kim, Yosef Koronyo, Maya Koronyo-Hamaoui, Foudil Lamari, Todd Langevin, Stéphane Lehéricy, Simone Lista, Jean Lorenceau, Mark Mapstone, Christian Neri, Robert Nisticò, Francis Nyasse-Messene, Sid E. O’bryant, George Perry, Craig Ritchie, Katrine Rojkova, Simone Rossi, Amira Saidi, Emiliano Santarnecchi, Lon S. Schneider, Olaf Sporns, Nicola Toschi, Steven R. Verdooner, Andrea Vergallo, Nicolas Villain, Lindsay A. Welikovitch, Janet Woodcock, Erfan Younesi

**Affiliations:** 1 Department of Psychosomatic Medicine, Clinical Dementia Research, Faculty of Medicine, Rostock University, Rostock, Germany; 2 German Center for Neurodegenerative Diseases (DZNE), Rostock, Germany; 3 AXA Research Fund and Sorbonne University Chair, Paris, France; 4 Sorbonne University, GRC n° 21, Alzheimer Precision Medicine (APM), AP-HP, Pitié-Salpêtrière Hospital, Boulevard de l’hôpital, F-75013 Paris, France; 5 Brain and Spine Institute (ICM), INSERM U 1127, CNRS UMR 7225, Boulevard de l’hôpital, F-75013 Paris, France; 6 Department of Neurology, Institute of Memory and Alzheimer’s Disease (IM2A), Pitié-Salpêtrière Hospital, AP-HP, Boulevard de l’hôpital, F-75013 Paris, France; 7 Qynapse, Paris, France; 8 Sorbonne University, UPMC University Paris 06, CNRS, INSERM, Laboratoire d’Imagerie Biomédicale, F-75013 Paris, France; 9 Multi-center Neuroimaging Platform, https://www.cati-neuroimaging.com; 10 Department of Nuclear Medicine, Pitié-Salpêtrière Hospital, AP-HP, F-75013 Paris, France; 11 Department of Neurology, University Medical Center of the Johannes-Gutenberg-University Mainz, Langenbeck str, 155131 Mainz, Germany; 12 Centre de NeuroImagerie de Recherche (CENIR), Institut du Cerveau et de la Moelle Epiniere (ICM), Paris, France; 13 Department of Neuroradiology, Salpêtriere Hospital, Paris, France

**Correction: Alz Res Therapy 11, 15 (2019)**

**https://doi.org/10.1186/s13195-019-0466-3**

Following the publication of the original article [1] the authors reported that members of “Alzheimer Precision Medicine Initiative (APMI)” are not visible in the collaborator list indexed.

The original article [1] has been updated.

Below are members of **the Alzheimer Precision Medicine Initiative Working Group (APMI–WG):**

Lisi Flores Aguilar, Claudio Babiloni, Filippo Baldacci, Norbert Benda, Keith L. Black, Arun L.W. Bokde, Ubaldo Bonuccelli, Karl Broich, René S. Bun, Francesco Cacciola, Juan Castrillo, Enrica Cavedo, Roberto Ceravolo, Patrizia A. Chiesa, Olivier Colliot, Cristina-Maria Coman, Jean-Christophe Corvol, Augusto Claudio Cuello, Jeffrey L. Cummings, Herman Depypere, Bruno Dubois, Andrea Duggento, Stanley Durrleman, Valentina Escott-Price, Howard Federoff, Maria Teresa Ferretti, Massimo Fiandaca, Richard A. Frank, Francesco Garaci, Remy Genthon, Nathalie George, Filippo S. Giorgi, Manuela Graziani, Marion Haberkamp, Marie-Odile Habert, Harald Hampel, Karl Herholz, Eric Karran, Seung H. Kim, Yosef Koronyo, Maya Koronyo-Hamaoui, Foudil Lamari, Todd Langevin, Stéphane Lehéricy, Simone Lista, Jean Lorenceau, Mark Mapstone, Christian Neri, Robert Nisticò, Francis Nyasse-Messene, Sid E. O’bryant, George Perry, Craig Ritchie, Katrine Rojkova, Simone Rossi, Amira Saidi, Emiliano Santarnecchi, Lon S. Schneider, Olaf Sporns, Nicola Toschi, Steven R. Verdooner, Andrea Vergallo, Nicolas Villain, Lindsay A. Welikovitch, Janet Woodcock, Erfan Younesi
