# Supplementary figures and images for: Applicability of in vivo staging of regional amyloid burden in a cognitively normal cohort with subjective memory complaints: the INSIGHT-preAD study
Source: Alzheimers Res Ther. 2019 Jan 31;11:15. doi: 10.1186/s13195-019-0466-3 (PMC6357385; doi:10.1186/s13195-019-0466-3)

**Figure S1** : Schematic diagram summarizing the pre-processing pipeline

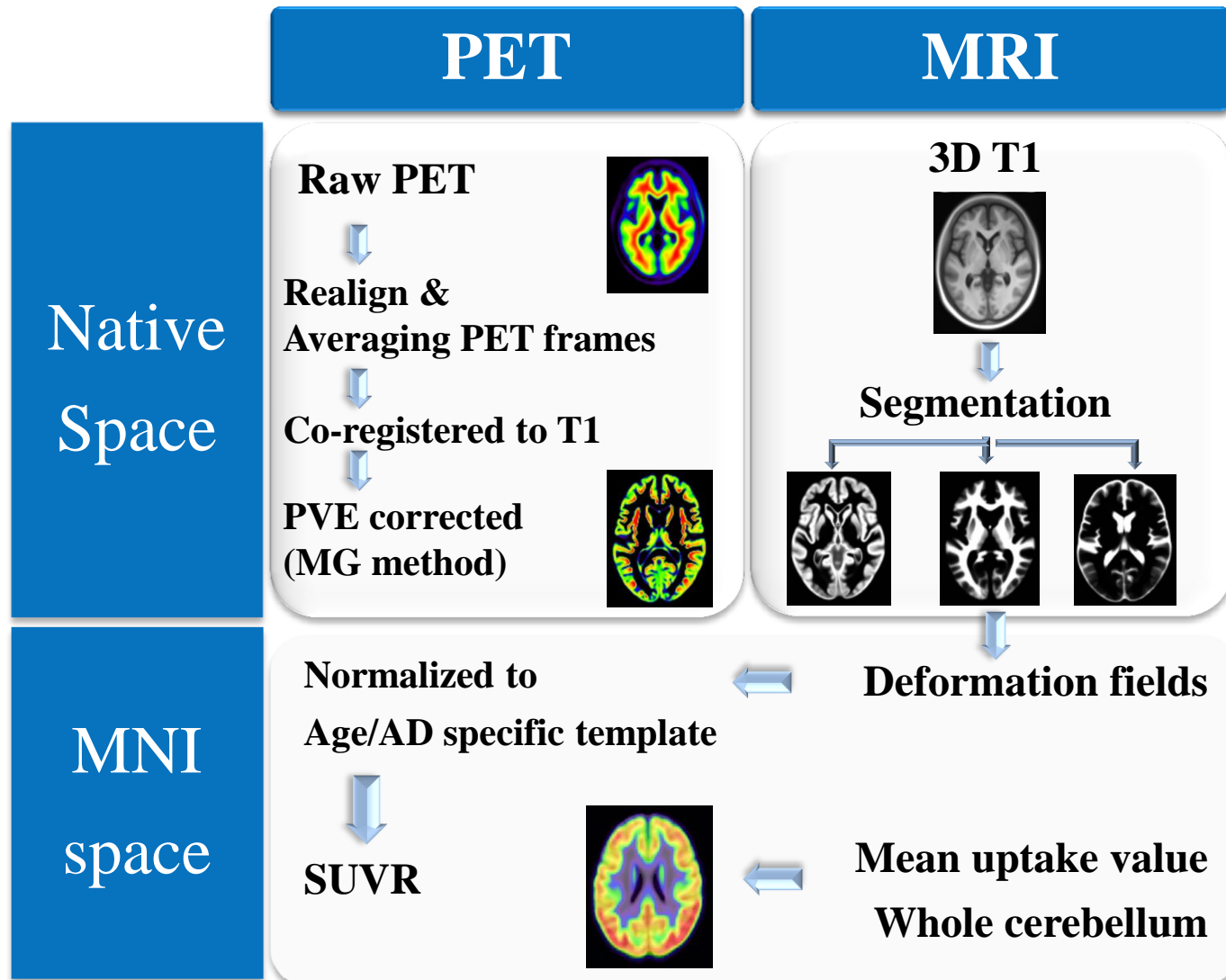

Supplement: Supplementary file 1 — Figure S1. Schematic diagram summarizing the pre-processing pipeline. (PDF 276 kb) [file 13195_2019_466_MOESM1_ESM.pdf]
