# Supplementary material for: Applicability of in vivo staging of regional amyloid burden in a cognitively normal cohort with subjective memory complaints: the INSIGHT-preAD study
Source: Alzheimers Res Ther. 2019 Jan 31;11:15. doi: 10.1186/s13195-019-0466-3 (PMC6357385; doi:10.1186/s13195-019-0466-3)
Supplement: Supplementary file 2 — Figure S2. Regional amyloid positivity cutoff value estimation. The figure shows the linear regression plot of the Global 18F-florbetapir PET SUVRcereb of both non-corrected (X-axis) and PVE-corrected PET (Y-axis) along with the generated equation that was used to transform the regional cutoff value of SUVRcereb = 1.135 to a value of SUVRcereb = 0.98 in the PVE-corrected PET data. (PDF 190 kb) [file 13195_2019_466_MOESM2_ESM.pdf]

Additional file 2

Figure S2 : Regional amyloid positivity cutoff value estimation.

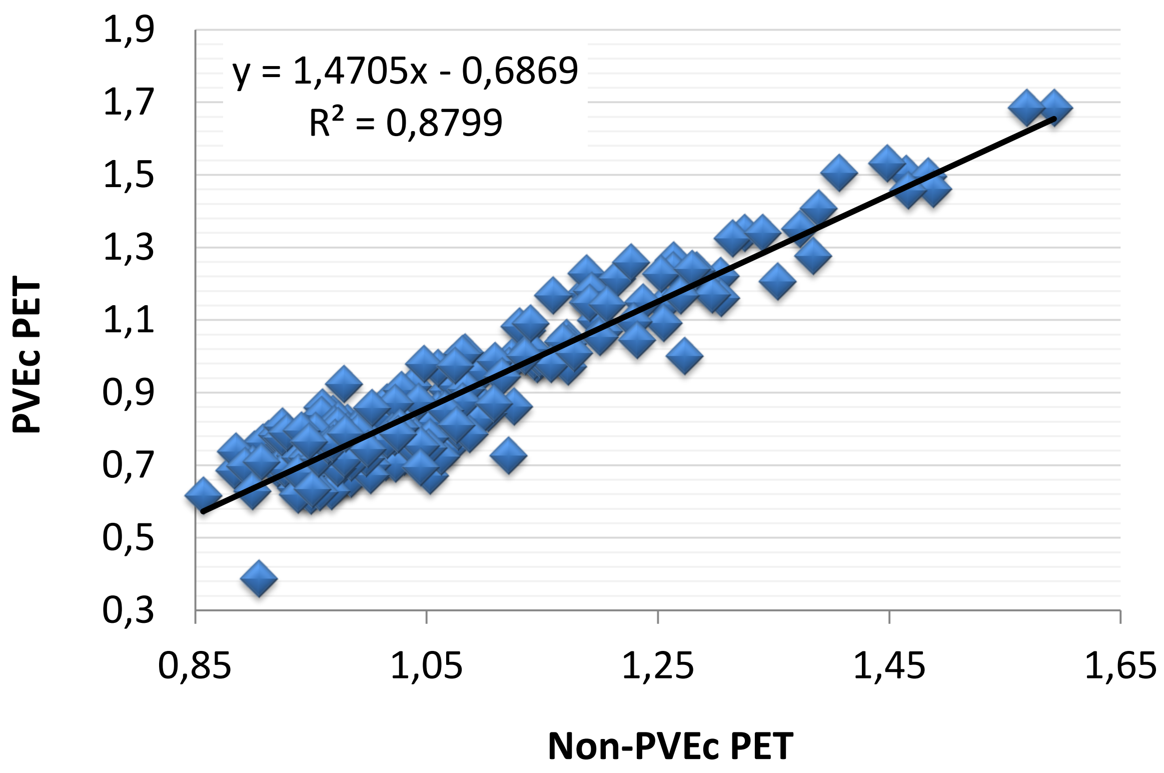

The figure shows the linear regression plot of the Global Florbetapir-PET SUVRcereb of both non-corrected (X-axis) and PVE-corrected PET (Y- axis) along with the generated equation that was used to transform the regional cutoff value of SUVRcereb = 1.135 to a value of SUVRcereb = 0.98 in the PVE-corrected PET data.
