# Supplementary material for: Applicability of in vivo staging of regional amyloid burden in a cognitively normal cohort with subjective memory complaints: the INSIGHT-preAD study
Source: Alzheimers Res Ther. 2019 Jan 31;11:15. doi: 10.1186/s13195-019-0466-3 (PMC6357385; doi:10.1186/s13195-019-0466-3)
Supplement: Supplementary file 3 — Figure S3. Model of the hierarchical in vivo amyloid staging scheme. This figure shows the 52 brain regions merged into four larger anatomical divisions based on equal partitions of frequency range as initially defined in the original model. Then the resulting amyloid progression stage (I-IV) is defined by the involvement of the corresponding anatomical division displayed in red in addition to the affected areas of the previous stage (displayed in blue). The amyloid progression stages are displayed on left, midline sagittal and basal brain views. (PDF 312 kb) [file 13195_2019_466_MOESM3_ESM.pdf]

Additional file 3

Figure S3 : Model of the hierarchical in-vivo amyloid staging scheme.

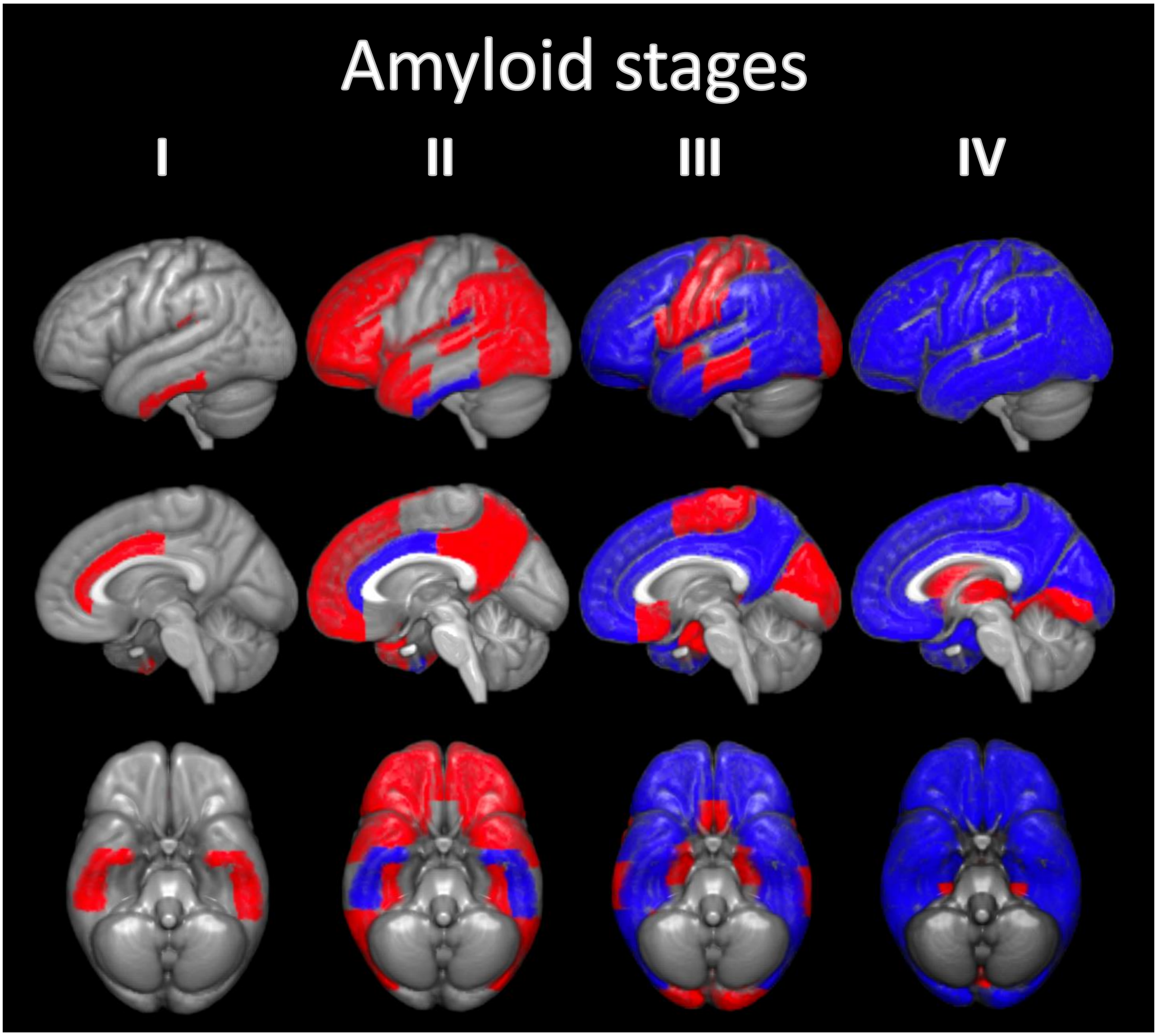

This figure shows the 52 brain regions merged into four larger anatomical divisions based on equal partitions of frequency range as initially defined in the original model. Then the resulting amyloid progression stage (I-IV) is defined by the involvement of the corresponding anatomical division displayed in red in addition to the affected areas of the previous stage (displayed in blue). The amyloid progression stages are displayed on left, midline sagittal and basal brain views.
