# Supplementary material for: Applicability of in vivo staging of regional amyloid burden in a cognitively normal cohort with subjective memory complaints: the INSIGHT-preAD study
Source: Alzheimers Res Ther. 2019 Jan 31;11:15. doi: 10.1186/s13195-019-0466-3 (PMC6357385; doi:10.1186/s13195-019-0466-3)
Supplement: Supplementary file 4 — Table S1. Principal component analysis applied on the neurocognitive test scores. The table shows the three main components that could be identified based on the principal component analysis and subsequently the contributing tests in each component. (PDF 97 kb) [file 13195_2019_466_MOESM4_ESM.pdf]

Additional file 4:

Table S1: Principal component analysis applied on the cognitive neuropsychiatric tests.

Rotated Components matrix[a]			
**Z-scores**	**Components**		
	**1**	**2**	**3**
FCSRT_total_free_recall	**0.939***	0.246	
FCSRT_free_recall3	0.829		
FCSRT_free_recall2	0.811	0.254	
FCSRT_delayed_free_recall	0.764	0.204	
FCSRT_free_recall1	0.755		
Verbal fluency Categories	0.426		0.329
_MCT_RI_List1+2	0.307	**0.917***	
_MCT_Binding_Score	0.297	0.910	
_MCT_immediate recall_List2	0.201	0.842	
MCT immediate recall _List1	0.250	0.652	
FCSRT_ immediate recall		0.315	0.305
TMT_B			**-0.721***
TMT_A			-0.604
Digitspan_backwards			0.524
Blockspan_forwards			0.478
Blockspan_backwards		0.282	0.471
ReyFig_copy			0.442
Digitspan_forwards			0.428
ReyFig_30min			0.400
ReyFig_3min			0.346
Verbal_fluency_lexical	0.321		0.324
TMT (B-A)			-0.216
**Explained Variance**	**18.5 %**	**14.9 %**	**12.06 %**

Extraction method: Main Components analysis.

Rotation method: Varimax with Kaiser-Normalisation.

a. The Rotation is in 5 Iterations converged.

The table shows the three main components that could be identified based on the principal component analysis and subsequently the contributing tests in each component. The cognitive test with the highest loadings in each of the three extracted principal components is written in bold and marked by the symbol (*).
