# Supplementary material for: Applicability of in vivo staging of regional amyloid burden in a cognitively normal cohort with subjective memory complaints: the INSIGHT-preAD study
Source: Alzheimers Res Ther. 2019 Jan 31;11:15. doi: 10.1186/s13195-019-0466-3 (PMC6357385; doi:10.1186/s13195-019-0466-3)
Supplement: Supplementary file 5 — Table S2. Associations between in vivo amyloid stage and cognitive performance. Analysis of Covariance (ANCOVA) assessing the effect of amyloid stage and conventional binary amyloid status on scores of the main principal components as well as the most representative tests for each of these components. (PDF 240 kb) [file 13195_2019_466_MOESM5_ESM.pdf]

**Additional file 5:**

**Table S2:** Associations between in-vivo amyloid stage and cognitive performance.

Analysis of Covariance (ANCOVA) assessing the effect of in-vivo amyloid stage and conventional binary amyloid status on scores of the main principal components as well as the most representative tests for each of these components. Effects for in-vivo amyloid stage were assessed both with and without inclusion of the few individuals classified as stage 4 (N=4). Binary global amyloid status was assessed using both standard non-PVE corrected data and PVE-corrected data. In the binary approach a standard cutoff of SUVR > 1.10 was used for non-PVE corrected data, and was for PVE-corrected data the same cutoff value was extrapolated to a value of SUVR = 0.93 (using the linear regression approach depicted in Additional File 2).

The values that passed the nominal level of significance ($p < 0.05$) are written in bold and marked by the symbol (*). None of these values survived Bonferroni correction for multiple comparisons across the 3 principal component scores and their most representative test scores (total of 6 statistical tests).

SS: Sum of squares; df: degree of freedom; MS: Mean squares; Sig: significance

| | Model | SS | df (error) | MS | F | Sig. (p value) | Partial $\eta^2$ |
|---|---|---|---|---|---|---|---|
| **PC1** | Staging4 (0.98) | 6.484 | 3(142) | 2.161 | 2.102 | 0.103 | 0.043 |
| | Staging(exclude stage4) | 5.083 | 2(139) | 2.542 | 2.467 | 0.089 | 0.034 |
| | Binary non –PVC-PET | 2.240 | 1(299) | 2.240 | 2.412 | 0.122 | 0.008 |
| | Binary PVC-PET | 2.177 | 1(299) | 2.177 | 2.342 | 0.127 | 0.008 |
| **PC2** | Staging4 (0.98) | 1.280 | 3(142) | 0.427 | 0.431 | 0.731 | 0.009 |
| | Staging(exclude stage4) | 0.561 | 2(139) | 0.280 | 0.296 | 0.744 | 0.004 |
| | Binary non –PVC-PET | 0.117 | 1(299) | 0.117 | 0.118 | 0.732 | 0.0001 |
| | Binary PVC-PET | 0.076 | 1(299) | 0.076 | 0.077 | 0.781 | 0.0001 |
| **PC3** | Staging4 (0.98) | 10.198 | 3(142) | 3.399 | 4.463 | **0.005*** | **0.086*** |
| | Staging(exclude stage4) | 3.383 | 2(139) | 1.692 | 2.202 | 0.114 | 0.031 |
| | Binary non –PVC-PET | 0.002 | 1(299) | 0.002 | 0.003 | 0.960 | 0.0001 |
| | Binary PVC-PET | 0.067 | 1(299) | 0.067 | 0.075 | 0.785 | 0.0001 |

| | Model | SS | df (error) | MS | F | Sig. (p value) | Partialη$^2$ |
|---|---|---|---|---|---|---|---|
| FCSRT total free recall | Staging4 (0.98) | 10.022 | 3(147) | 3.341 | 3.292 | **0.022*** | **0.063*** |
| | Staging(exclude stage4) | 5.455 | 2(144) | 2.728 | 2.661 | 0.073 | 0.030 |
| | Binary non –PVC-PET | 2.921 | 1(313) | 2.921 | 3.146 | 0.077 | 0.010 |
| | Binary PVC-PET | 2.449 | 1(313) | 2.449 | 2.633 | 0.106 | 0.008 |
| MCT- Immediate Free recall (List A+B) | Staging4 (0.98) | 1.836 | 3(143) | 0.612 | 0.661 | 0.578 | 0.014 |
| | Staging(exclude stage4) | 0.921 | 2(140) | 0.460 | 0.515 | 0.599 | 0.007 |
| | Binary non –PVC-PET | 0.176 | 1(303) | 0.176 | 0.180 | 0.672 | 0.001 |
| | Binary PVC-PET | 0.039 | 1(303) | 0.039 | 0.40 | 0.842 | 0.0001 |
| TMT - B | Staging4 (0.98) | 8.607 | 3(147) | 2.869 | 2.922 | **0.036*** | **0.056*** |
| | Staging(exclude stage4) | 2.717 | 2(144) | 1.358 | 1.365 | 0.259 | 0.019 |
| | Binary non –PVC-PET | 1.040 | 1(311) | 1.040 | 1.114 | 0.292 | 0.004 |
| | Binary PVC-PET | 1.520 | 1(311) | 1.520 | 1.631 | 0.203 | 0.005 |

The median values of the cognitive tests scores with the highest loadings for each of the extracted principal components,(TMT B-scores, FCSRT total free recall scores and MCT both lists immediate recall scores, are shown for the different in-vivo amyloid stages.

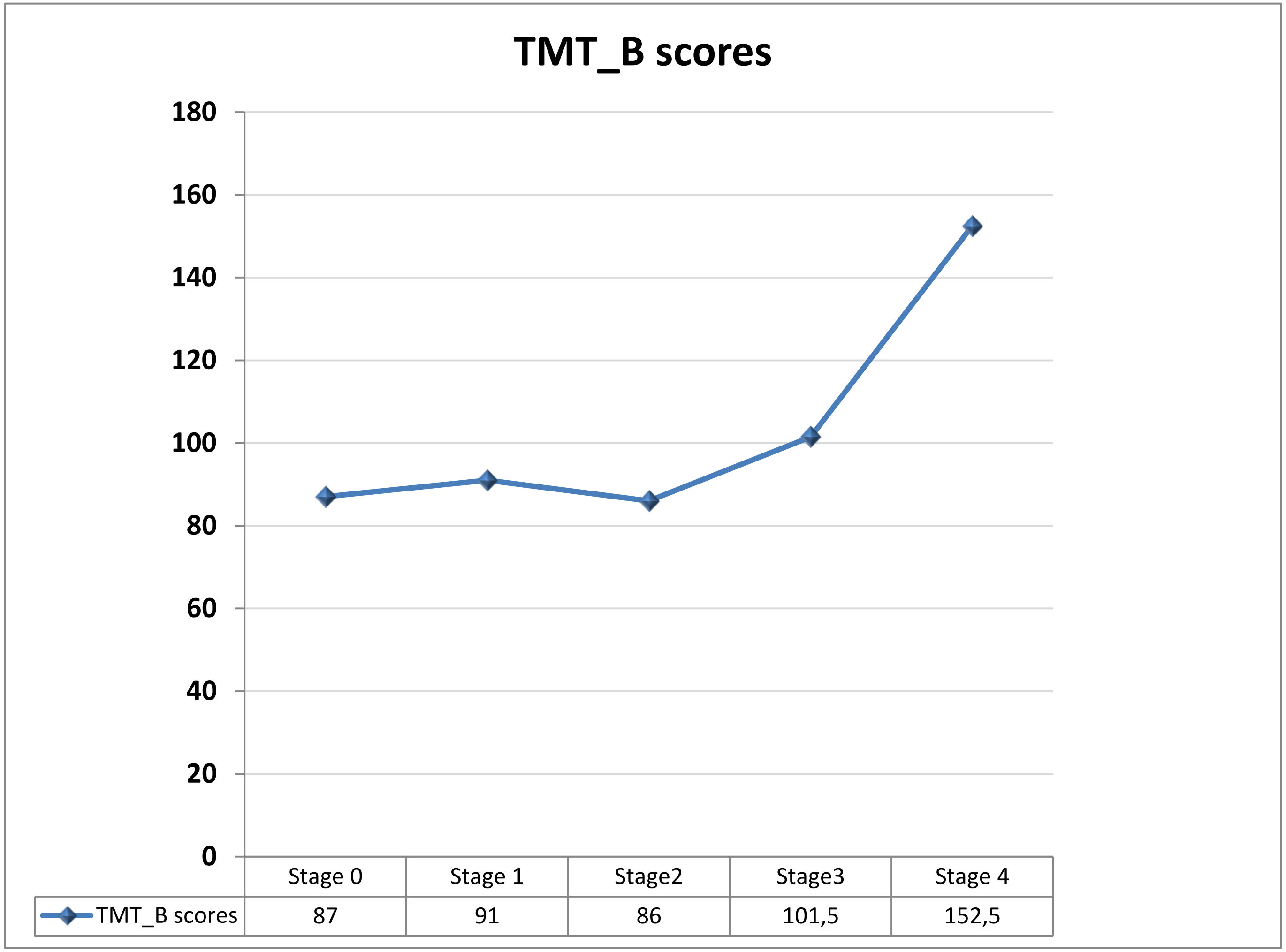

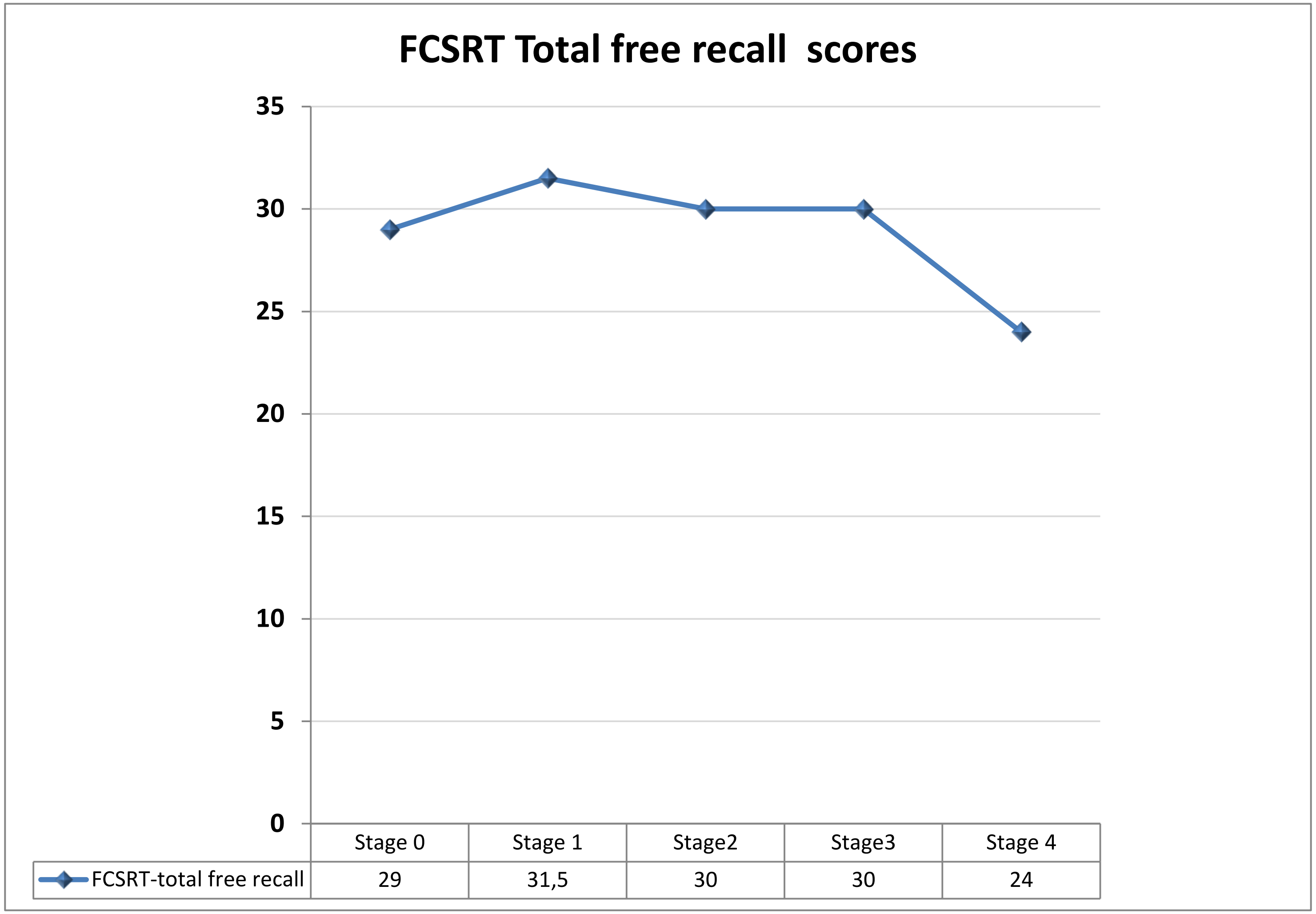
FCSRT Total free recall scores
35
30
25
20
15
10
5
0
Stage 0
Stage 1
Stage2
Stage3
Stage 4
FCSRT-total free recall
29
31,5
30
30
24

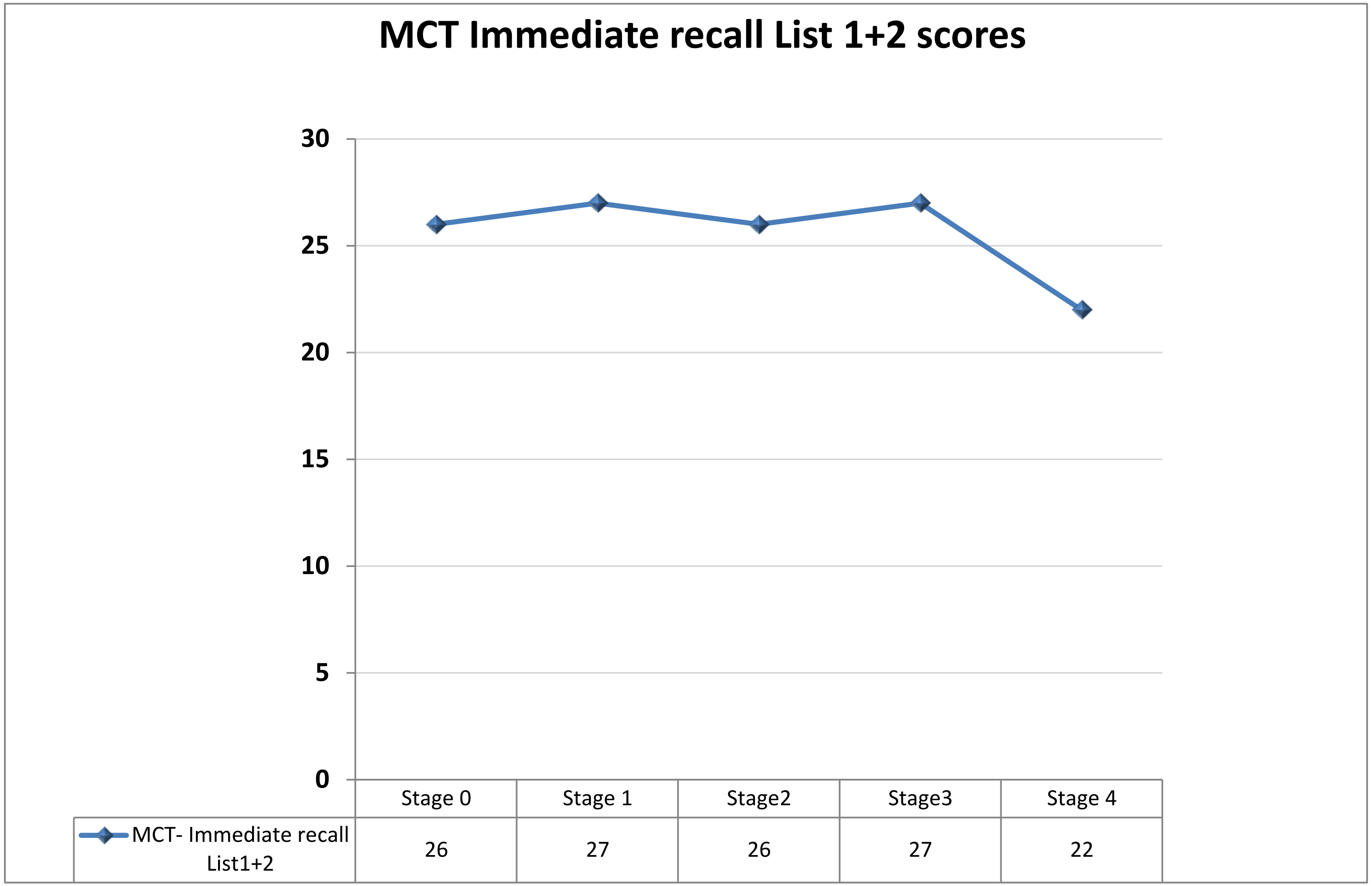
MCT Immediate recall List 1+2 scores
30
25
20
15
10
5
0
Stage 0
Stage 1
Stage2
Stage3
Stage 4
MCT- Immediate recall List1+2
26
27
26
27
22
