# Supplementary material for: Applicability of in vivo staging of regional amyloid burden in a cognitively normal cohort with subjective memory complaints: the INSIGHT-preAD study
Source: Alzheimers Res Ther. 2019 Jan 31;11:15. doi: 10.1186/s13195-019-0466-3 (PMC6357385; doi:10.1186/s13195-019-0466-3)
Supplement: Supplementary file 6 — Figure S4. Amyloid progression model in the INSIGHT-preAD data. This figure shows the amyloid progression model in the INSIGHT-preAD data as implied by the frequency of involvement of the 52 studied brain regions. The frequencies were displayed on left, midline sagittal and basal brain views. (PDF 252 kb) [file 13195_2019_466_MOESM6_ESM.pdf]

Additional file 6

Figure S4 : Amyloid progression model in the INSIGHT-preAD data.

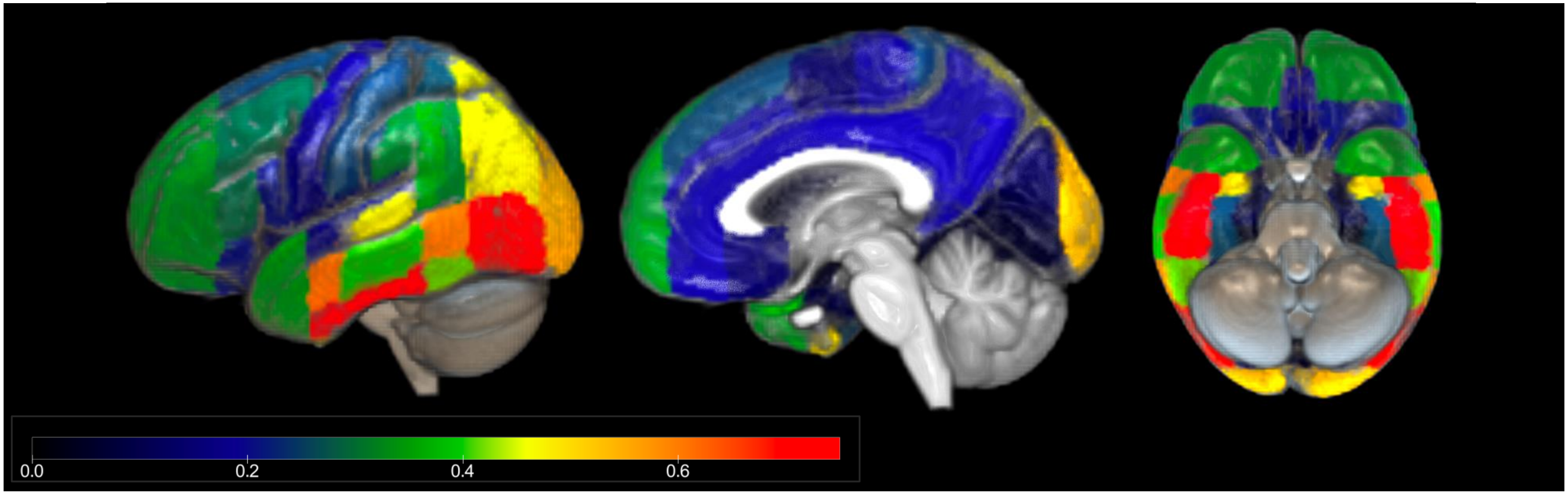

This figure shows the amyloid progression model in the INSIGHT-preAD data as implied by the frequency of involvement of the 52 studied brain regions. The frequencies were displayed on left, midline sagittal and basal brain views.
